# A combination of a cell penetrating peptide and a protein translation inhibitor kills metastatic breast cancer cells

**DOI:** 10.1038/s41420-023-01627-3

**Published:** 2023-08-31

**Authors:** Linda Rowland, Henri-Baptiste Marjault, Ola Karmi, DeAna Grant, Lauren J. Webb, Assaf Friedler, Rachel Nechushtai, Ron Elber, Ron Mittler

**Affiliations:** 1 Department of Surgery, University of Missouri School of Medicine, Christopher S. Bond Life Sciences Center University of Missouri, 1201 Rollins Street, Columbia, MO 65201 USA; 2 The Alexander Silberman Institute of Life Science, The Hebrew University of Jerusalem, Edmond J. Safra Campus at Givat Ram, Jerusalem, 9190401 Israel; 3 Electron Microscopy Core Facility, University of Missouri, 0011 NextGen Precision Health Institute, 1030 Hitt Street, Columbia, MO 65211 USA; 4 Department of Chemistry, The University of Texas at Austin, 2506 Speedway STOP A5300, Austin, TX 78712 USA; 5 Institute of Chemistry, The Hebrew University of Jerusalem, Edmond J. Safra Campus at Givat Ram, Jerusalem, 9190401 Israel; 6 Institute for Computational Engineering and Science and Department of Chemistry, University of Texas at Austin, Austin, TX 78712 USA

**Keywords:** Drug development, Breast cancer

## Abstract

Cell Penetrating Peptides (CPPs) are promising anticancer and antimicrobial drugs. We recently reported that a peptide derived from the human mitochondrial/ER membrane-anchored NEET protein, Nutrient Autophagy Factor 1 (NAF-1; NAF-1^44-67^), selectively permeates and kills human metastatic epithelial breast cancer cells (MDA-MB-231), but not control epithelial cells. As cancer cells alter their phenotype during growth and metastasis, we tested whether NAF-1^44–67^ would also be efficient in killing other human epithelial breast cancer cells that may have a different phenotype. Here we report that NAF-1^44–67^ is efficient in killing BT-549, Hs 578T, MDA-MB-436, and MDA-MB-453 breast cancer cells, but that MDA-MB-157 cells are resistant to it. Upon closer examination, we found that MDA-MB-157 cells display a high content of intracellular vesicles and cellular protrusions, compared to MDA-MB-231 cells, that could protect them from NAF-1^44–67^. Inhibiting the formation of intracellular vesicles and dynamics of cellular protrusions of MDA-MB-157 cells, using a protein translation inhibitor (the antibiotic Cycloheximide), rendered these cells highly susceptible to NAF-1^44–67^, suggesting that under certain conditions, the killing effect of CPPs could be augmented when they are applied in combination with an antibiotic or chemotherapy agent. These findings could prove important for the treatment of metastatic cancers with CPPs and/or treatment combinations that include CPPs.

## Introduction

Cell Penetrating Peptides (CPPs) are small peptides that penetrate the plasma membrane (PM) of cells via energy dependent (endocytosis) or energy independent (direct permeation) mechanisms [1, 2]. They are often used in medicine as cargo carriers of other drugs into cells, and/or as direct cell killers. Several different CPPs have been successfully used as antimicrobial or anticancer drugs, and CPPs in general are considered a promising avenue of therapy for numerous diseases and conditions [3, 4]. CPPs are sometimes derived from the membrane translocation machinery of proteins, or from naturally occurring antimicrobial peptides. Examples include the antimicrobial peptide buforin IIb that shows significant anticancer activity, the Membrane Targeting Peptide NeuNT (MTP-NeuNT) that disrupts the tyrosine kinase epidermal growth factor receptor ErbB2 dimer killing cancer cells, and the p53(15)Ant peptide, derived from p53 [5–7]. Another group of anticancer peptides are the naturally occurring antimicrobial Pore-Forming Peptides (PFPs) [8], that assemble on the PM of cells generating pores and killing cancer cells; however, without considerable specificity.

For CPPs or PFPs to be efficient as anticancer drugs, they must demonstrate a high degree of specificity, targeting and killing cancer cells without affecting healthy cells. Both hybrid and combination CPP drug strategies [9–11], as well as CPP-nanoparticle drug combination strategies [12–14] were used to increase specificity and lethality of CPPs. However, more studies that refine our understanding of CPP’s mode of function, and/or propose novel and/or targeted avenues for their delivery, are needed [1–4].

We recently reported that a peptide derived from the human mitochondrial- and ER membrane-anchored NEET protein, Nutrient Autophagy Factor 1 (NAF-1; CISD2), containing an N-terminal transmembrane hydrophobic segment and a charged C-terminal part (NAF-1^44–67^), selectively kills epithelial breast cancer cells without affecting control epithelial cells [15]. NAF-1^44–67^ selectively permeates through the PM of metastatic human epithelial breast cancer cells (MDA-MB-231), but not control epithelial cells (MCF-10A) and targets the mitochondria and ER membranes of cancer cells causing the release of cytochrome c and the activation of apoptotic and ferroptotic cell death pathways (only in cancer cells) [15]. This effect is most likely linked to the function of NAF-1 in regulating multiple apoptotic/ferroptotic/autophagy pathways in cells, and its interactions with BCL-2 family members, that are disrupted in cancer cells upon treatment with the peptide [15]. We further reported that NAF-1^44–67^ specifically permeates cancer cells via a direct permeation (i.e., energy independent) mechanism [15, 16], and that it significantly decreases tumor growth in an in vivo xenograft mice model system of human triple-negative breast cancer MDA-MB-231 tumors; without causing negative side effects to control healthy mice [15].

As cancer cells may alter their phenotype during growth and/or metastasis (e.g., the Epithelial-Mesenchymal Transition; EMT transition) [17, 18], and/or display large variability in PM composition, even within the same sub group of cancers (e.g., [19, 20]), we wanted to test whether NAF-1^44–67^, that permeates cancer cells via a direct PM permeation mechanism (that could depend on membrane composition, volume, or structure [15, 16]), would also be efficient in killing other human epithelial breast cancer cells that may have a different phenotype, membrane structures, or membrane composition, compared to MDA-MB-231 [19]. Here we report that NAF-1^44–67^ is efficient in killing BT-549, Hs 578T, MDA-MB-436, and MDA-MB-453 cancer cells, but that MDA-MB-157 cells are resistant to it. We further reveal that MDA-MB-157 cells display a high content of intracellular vesicles and cellular protrusions, compared to MDA-MB-231 cells, that could serve as a physical barrier against NAF-1^44–67^. Inhibiting the formation of intracellular vesicles and the dynamics of cellular protrusions of MDA-MB-157 cells, using an antibiotic protein translation inhibitor (i.e., Cycloheximide), rendered MDA-MB-157 cells highly susceptible to the peptide, suggesting that, under certain conditions, the killing effect of certain types of CPPs could be augmented when they are applied in combination with specific antibiotic or chemotherapy drugs that target membrane composition, structure, turnover, and/or function.

## Results

### Susceptibility of different epithelial breast cancer cell lines to NAF-1^44–67^

**Fig. 1:**
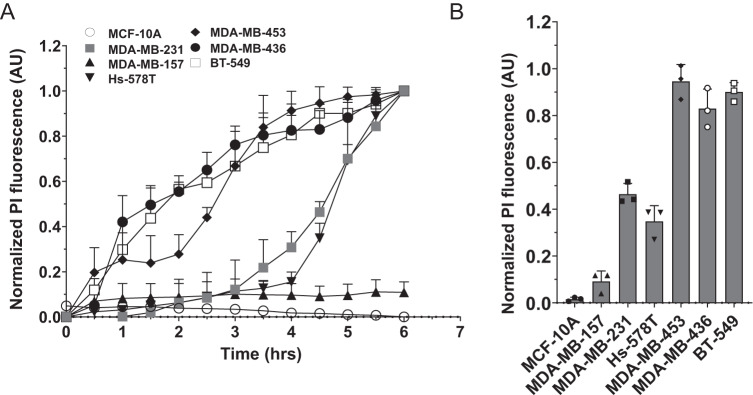
Susceptibility of triple-negative epithelial breast cancer cell lines to the NAF-1^44–67^ peptide. **A** Time course line graph analysis of cell death induction measured with propidium iodide (PI) in response to treatment with 15 μM of NAF-1^44–67^. **B** Bar graphs showing statistical analysis of cell death induced by the peptide at 5 h post peptide application. Detailed description of the different cancer cell lines used in the figure is shown in Table S1. The different cancer cell lines are compared to the control epithelial breast cell line MCF-10A. All experiments were repeated at least 3 times with 3 different technical repeats per cell line. Statistical significance was determined using one-way ANOVA. NAF-1 nutrient autophagy factor 1, PI propidium iodide, AU arbitrary units.

NAF-1^44–67^ was previously shown to kill MDA-MB-231 cells without affecting control MCF-10A cells [15]. To determine the effect of NAF-1^44–67^ on other human epithelial breast cancer cell lines that may have a different appearance or membrane composition [19, 20], we obtained the epithelial breast cancer cell lines shown in Table S1 from ATCC (https://www.atcc.org/), and compared their survival in the presence or absence of NAF-1^44–67^ (15 μM) to that of MCF-10A and MDA-MB-231 (Fig. 1). MDA-MB-436 (a primary carcinoma cell line), and BT-549 and MDA-MB-453 (metastatic cell lines), displayed the highest susceptibility to NAF-1^44–67^, while Hs-578T (a primary carcinoma cell line), was about as sensitive as metastatic MDA-MB-231 to NAF-1^44–67^ (Fig. 1). In contrast, the metastatic cell line MDA-MB-157 appeared to be resistant to NAF-1^44–67^ and displayed a similar response to the peptide as control MCF-10A cells (Fig. 1). These results suggest that the MDA-MB-157 cell line is resistant to NAF-1^44–67^.

### Dose-dependent effect of NAF-1^44–67^ on MDA-MB-231, MCF-10A, and MDA-MB-157

**Fig. 2:**
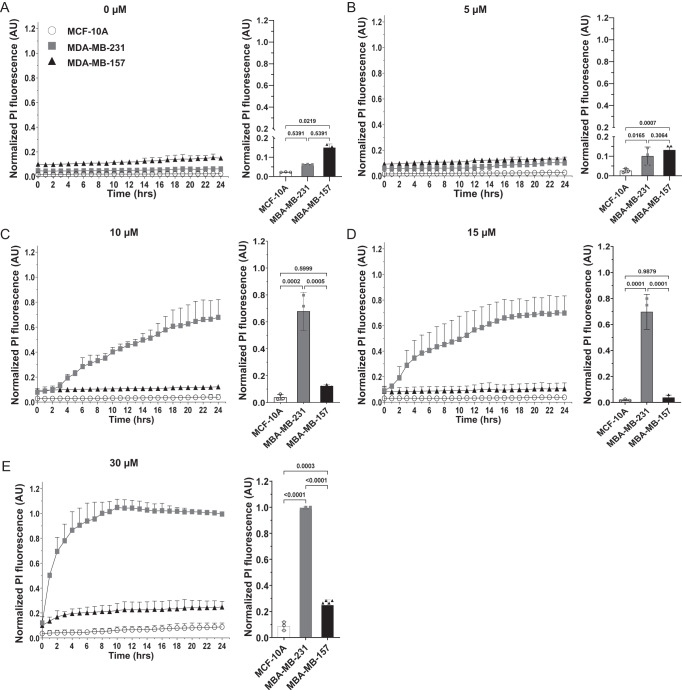
Dosage effect of the NAF-1^44–67^ peptide on MCF-10A, MDA-MB-231, and MDA-MB-157 cells. **A**–**E** Time course line graph analyses (left) and bar graphs showing statistical analyses of cell death at 24 h (right) induced in MCF-10A, MDA-MB-231 and MDA-MB-157, by the application of 0 (**A**), 5 (**B**), 10 (**C**), 15 (**D**), and 30 (**E**) μM of NAF-1^44–67^. All experiments were repeated at least 3 times with 3 different technical repeats per cell line. Statistical significance was determined using one-way ANOVA. NAF-1 nutrient autophagy factor 1, PI propidium iodide, AU arbitrary units.

To further study the response of MDA-MB-157 to NAF-1^44–67^, we focused on this cell line and compared its resistance to different concentrations of NAF-1^44–67^, applied over a longer time period (0–50 h, Fig. 2, as opposed to 6 h shown in Fig. 1). Application of 0 or 5 μM of NAF-1^44–67^ did not cause a significant increase in cell death of MDA-MB-231, MCF-10A, or MDA-MB-157 (Fig. 2A, B). In contrast, application of 10 or 15 μM NAF-1^44–67^ caused cell death in the MDA-MB-231 cell line without affecting MCF-10A or MDA-MB-157 (Fig. 2C, D). Application of 30 μM NAF-1^44–67^ caused some level of cell death in MDA-MB-157, that was higher after 10 h of exposure, compared to MCF-10A, but still much lower compared to MDA-MB-231 (Fig. 2E). The findings shown in Fig. 2, suggest that the resistance of MDA-MB-157 to NAF-1^44–67^ could be partially overcome using a higher concentration of the peptide. Considering the energy-independent mode of NAF-1^44–67^ entry into cancer cells [15, 16], this finding could suggest that compared to MDA-MB-231, or the other epithelial breast cancer cell lines tested (Fig. 1), the PM of MDA-MB-157 has a higher ability to block the peptide from entering cells, and/or retain the peptide in vesicles/membrane structures inside or outside MDA-MB-157, preventing it from reaching the cytosol and mitochondria.

### Phenotype of MDA-MB-231, MCF-10A, and MDA-MB-157 cells

**Fig. 3:**
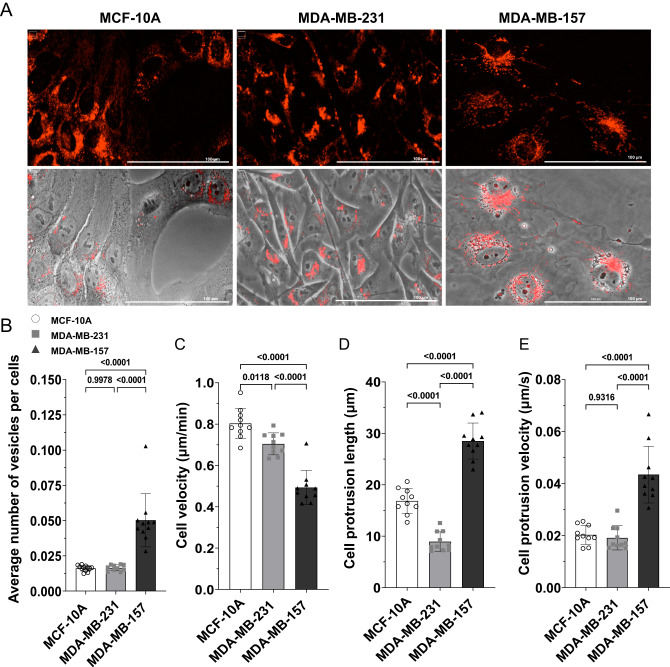
Phenotypic characterization of MCF-10A, MDA-MB-231, and MDA-MB-157 cells growing in the absence of the peptide. **A** Representative images of the different cell lines, imaged with the plasma membrane (PM) fluorescent dye SynaptoRed^TM^ C2 (top), or with the PM dye and phase contrasting (bottom). **B**–**E** Bar graphs showing the average number of vesicles per cell (**B**), cell velocity (**C**), length of cell protrusions (**D**), and rate of cell protrusion formation (**E**). All experiments were repeated at least 3 times with 3 different technical repeats per cell line (each containing 50 different microscopic images). Statistical significance was determined using one-way ANOVA. PM plasma membrane.

The membrane composition of the MDA-MB-157 cell line was previously found to be different than that of MDA-MB-231 [19]. However, the membrane composition of several other cell lines, that were susceptible to NAF-1^44–67^ (Fig. 1 and Table S1) is also different from that of MDA-MB-231 [19, 21–24], suggesting that membrane composition might not be a major player in determining susceptibility to NAF-1^44–67^. We therefore focused on structural differences between the different cell lines that could explain the resistance of MDA-MB-157 cells to NAF-1^44–67^ (Fig. 3). This analysis revealed that compared to MCF-10A or MDA-MB-231, MDA-MB-157 cells contained a higher number of intracellular vesicles (Fig. 3A, B). Although, MDA-MB-157 cells had a slower mobility compared to MCF-10A or MDA-MB-231 (Fig. 3C), they displayed longer and more frequent protrusions that appeared to exchange membrane and/or other cellular material between cells (Fig. 3D, E and Movies S1–S3). This phenotype suggested that MDA-MB-157 cells could have a more dynamic metabolic, cytoskeleton, and/or membrane synthesis activity(s), compared to MCF-10A or MDA-MB-231, and/or contained a higher volume of overall membrane material (Fig. 3 and Movies S1–S3). The findings presented in Fig. 3 and Movies S1–S3 suggest that the resistance of MDA-MB-157 to NAF-1^44–67^ could result from a higher turnover rate of membranes and/or the formation of vesicles and/or cellular protrusions that absorb the peptide and do not allow it to enter the cytosol. This possibility is in agreement with the findings that a high concentration of the peptide (30 μM) was able to decrease the survival of MDA-MB-157 cells (Fig. 2E), potentially a result of overcoming the physical membrane barriers of MDA-MB-157 cells (Fig. 3 and Movies S1–S3) that prevent the peptide from entering and killing it.

### The effect of NAF-1^44–67^ on the mitochondria of MDA-MB-231, MCF-10A, and MDA-MB-157 cells

**Fig. 4:**
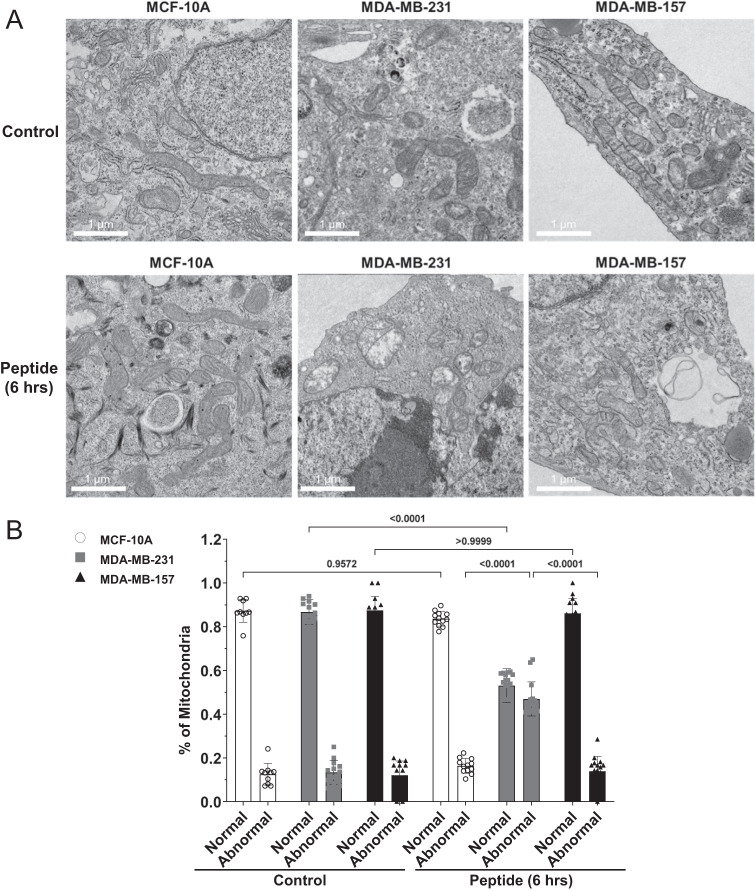
Transmission electron microscopy (TEM) analysis of mitochondrial damage induced in MCF-10A, MDA-MB-231, and MDA-MB-157 cells by the NAF-1^44–67^ peptide (15 μM). **A** representative TEM images of mitochondria from the different cell lines in the absence or presence of the NAF-1^44–67^ peptide. **B** Statistical analysis of the amount of normal or abnormal mitochondria in the different cell lines under control conditions or following treatment with 15 μM of the NAF-1^44–67^ peptide. Abnormal mitochondria were defined as swollen mitochondria with damaged or missing crista (damaged mitochondria). All experiments were repeated at least 3 times with 3 different technical repeats per cell line (each containing 50 different microscopic images). Statistical significance was determined using one-way ANOVA. NAF-1 nutrient autophagy factor 1.

A hallmark of the cancer killing activity of NAF-1^44–67^ towards MDA-MB-231 cancer cells is the extensive structural damage it causes their mitochondria; that is absent in control MCF-10A cells [15]. To test whether the failure of NAF-1^44–67^ to kill MDA-MB-157 was reflected in the degree of mitochondrial damage induced in these cells by the peptide, we compared the structure of mitochondria observed by transmission electron microscopy (TEM) analysis between treated and untreated MCF-10A, MDA-MB-231, and MDA-MB-157 cells (Fig. 4). Compared to the destructive impact of NAF-1^44–67^ (15 μM) on the mitochondria of MDA-MB-231 cells, the peptide had no apparent effect on the mitochondria of MCF-10A or MDA-MB-157 cells (Fig. 4A, B), further supporting the possibility that it is unable to enter the cytosol of MDA-MB-157 cells at this concentration (Figs. 2–4).

### Binding of NAF-1^44–67^ to the plasma membrane of MDA-MB-231, MCF-10A, and MDA-MB-157 cells

**Fig. 5:**
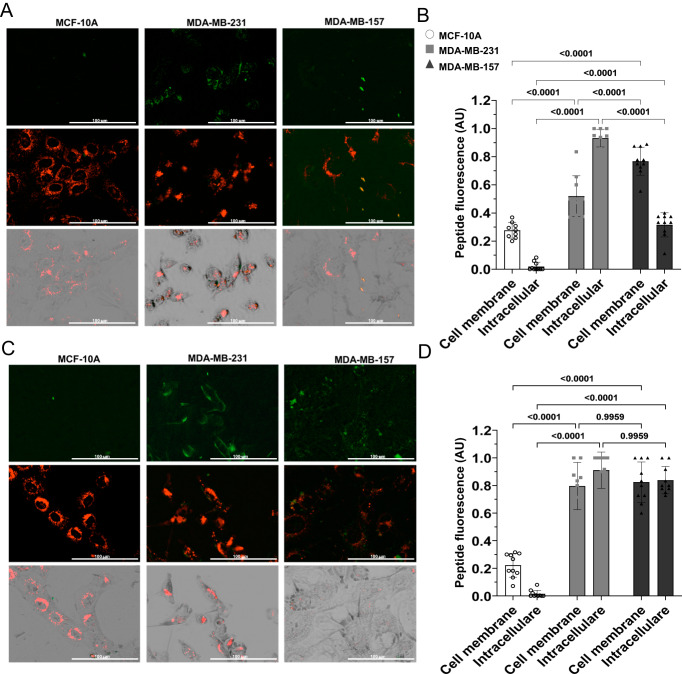
Binding of the NAF-1^44–67^ peptide to the membranes of MCF-10A, MDA-MB-231, and MDA-MB-157 cells in the presence or absence of cycloheximide. **A** Representative images of the different cell lines treated with a 5(6)-carboxyfluorescein-labeled peptide (Fl-NAF-1^44–67^; N-termini; 15 μM). Top panels show fluorescein fluorescence, middle panels show plasma membrane (PM) fluorescent dye SynaptoRed^TM^ C2 staining, and bottom panels show a merge of the top two with phase contrasting. **B** Bar graphs and statistical analyses showing the amount of fluorescence (Fl-NAF-1^44–67^) at the cell membrane or intracellularly, in the different cell lines. **C**, **D** Same as **A**, **B** but in the presence of 100 μM cycloheximide. All experiments were repeated at least 3 times with 3 different technical repeats per cell line (each containing 50 different microscopic images). Statistical significance was determined using one-way ANOVA. NAF-1 nutrient autophagy factor 1, Fl fluorescein, PM plasma membrane, AU arbitrary units.

The findings presented in Figs. 2–4 suggest that NAF-1^44–67^ is unable to penetrate MDA-MB-157 cells when applied at a concentration of 15 μM. To directly test this possibility, we labeled the NAF-1^44–67^ peptide with fluorescein at its N-terminal and incubated the labeled peptide with MDA-MB-231, MCF-10A, and MDA-MB-157 cells for 30 min. We then quantified the amount of labeled peptide found on the PM or inside cells [15]. While labeled NAF-1^44–67^ could be found inside, and attached to, the PM of MDA-MB-231 cells (as previously reported [15]), it was primarily detected attached to the PM of MDA-MB-157 cells (Fig. 5A, B). Although some peptide could be detected inside of MDA-MB-157 cells, the levels of NAF-1^44–67^ found inside MDA-MB-157 cells were much lower compared to those found inside MDA-MB-231 cells (Fig. 5A, B). Because the peptide did not induce damage to the mitochondria of MDA-MB-157 cells (Fig. 4), it is possible that the peptide found inside of MDA-MB-157 cells was attached to vesicles, of PM origin (Fig. 3B). As previously reported [15], the labeled NAF-1^44–67^ peptide was not detected inside of MCF-10A cells (Fig. 5A). Taken together, the results shown in Figs. 2–5A suggest that, in contrast to MDA-MB-231, NAF-1^44–67^ (at a concentration of 15 μM) is unable to penetrate the PM of MDA-MB-157 cells, potentially due to the high membrane turnover/vesicle/protrusions activity of these cells (Fig. 4 and Movies S1–S3).

### A combined treatment of MDA-MB-157 cells with NAF-1^44–67^ and Cycloheximide decreases the vesicle content and cellular protrusions of MDA-MB-157 cells and renders them susceptible to treatment with a low dose of NAF-1^44–67^

**Fig. 6:**
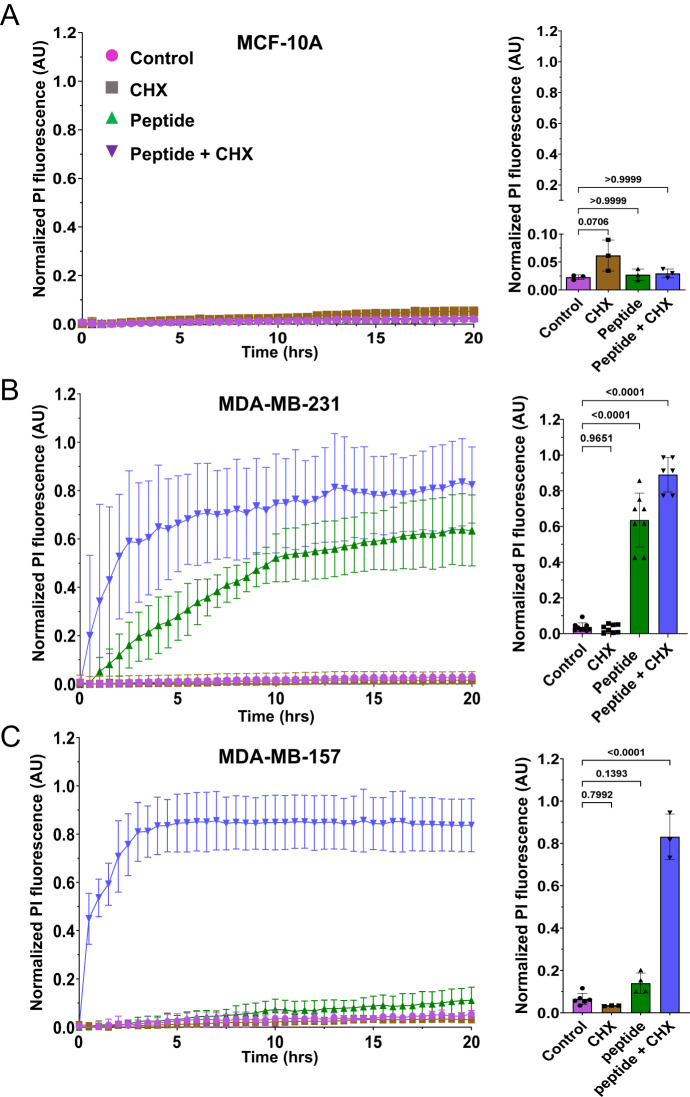
The effect of NAF-1^44–67^, cycloheximide (CHX), or NAF-1^44–67^ + CHX on cell death of the MCF-10A, MDA-MB-231, and MDA-MB-157 cell lines. **A**–**C** Time course line graph analyses (left) and bar graphs showing statistical analyses of cell death at 20 h (right) induced in MCF-10A (A), MDA-MB-231 (**B**) and MDA-MB-157 (**C**), by the application of NAF-1^44–67^, CHX, or NAF-1^44–67^ + CHX. All experiments were repeated at least 3 times with 3 different technical repeats per cell line. Statistical significance was determined using one-way ANOVA. CHX cycloheximide, NAF-1 nutrient autophagy factor 1, PI propidium iodide, AU arbitrary units.

To test the possibility that MDA-MB-157 cells are evading NAF-1 peptide toxicity due to their enhanced membrane turnover/high vesicle content and/or the formation of protrusions, we treated MDA-MB-231, MCF-10A, and MDA-MB-157 cells with NAF-1^44–67^ (15 μM) in the presence or absence of the eukaryotic protein synthesis antibiotic inhibitor Cycloheximide (CHX; 100 μM). As opposed to untreated MDA-MB-157 cells, incubated with the labeled peptide (15 μM; Fig. 5A, B), the CHX treatment resulted in more of the peptide being detected inside of MDA-MB-157 cells (Fig. 5C, D); suggesting that CHX treatment can suppress the ability of MDA-MB-157 cells to block or delay the entry of the peptide into their cytosol (Figs. 1–5), thereby enhancing the killing effect of NAF-1^44–67^ (at 15 μM). To test this possibility, we measured cell death of MCF-10A, MDA-MB-231, and MDA-MB-157 in the presence or absence of NAF-1^44–67^, CHX, and NAF-1^44–67^ + CHX (Fig. 6 and Movies S4–S12).

While treatment of MDA-MB-157 cells with the peptide (15 μM), or CHX (100 μM), did not cause cells death, treatment of MDA-MB-157 cells with a combination of NAF-1^44–67^ (15 μM) + CHX (100 μM) caused these cells to die (Fig. 6C and Movies S4–S6). In contrast, treatment of MCF-10A with NAF-1^44–67^ (15 μM) and/or CHX (100 μM) did not result in enhanced cell death (Fig. 6A and Movies S7–S9), and treatment of MDA-MB-231 with the peptide in the presence or absence of CHX caused these cells to die (Fig. 6B and Movies S10–S12).

**Fig. 7:**
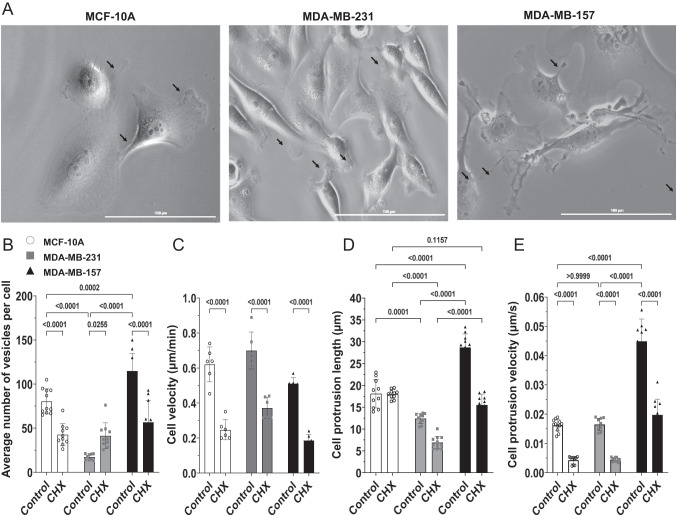
The effect of cycloheximide (CHX) on the phenotype of MCF-10A, MDA-MB-231, and MDA-MB-157 cells. **A** Representative images of the different cell lines imaged by phase contrasting. Arrows indicate cellular protrusions. **B**–**E** Bar graphs showing the effect of CHX (100 μM) on the average number of vesicles per cell (**B**), cell velocity (**C**), length of cell protrusions (**D**), and rate of cell protrusion formation (**E**). All experiments were repeated at least 3 times with 3 different technical repeats per cell line (each containing 50 different microscopic images). Statistical significance was determined using one-way ANOVA. CHX cycloheximide.

To test whether the treatment of MDA-MB-157 cells with CHX (100 μM) caused these cells to have fewer vesicles and/or produce less cellular protrusions, we studied the cell velocity, vesicle content, protrusion length, and rate of protrusion formation in MCF-10A, MDA-MB-231, and MDA-MB-157 cells in the presence or absence of CHX (100 μM; Fig. 7). This analysis revealed that cell velocity, vesicle content, protrusion length, and rate of protrusion formation were suppressed in all cell lines by the CHX treatment (Fig. 7 and Movies S1–S3, S5, S8, S11). Of particular importance was the observation that the suppression of protrusion length and rate of protrusion formation in MDA-MB-157 cells by CHX brought the activity level of these cells to that of untreated (and peptide susceptible; Figs. 1, 2, and 6) MDA-MB-231 cells (Fig. 7D, E). Taken together, the findings presented in Figs. 5–7 suggest that the effect of CHX on the susceptibility of MDA-MB-157 to NAF-1^44–67^ correlates with the effects of CHX on vesicle content, and rate of formation and length of cellular protrusion of these cells.

## Discussion

**Fig. 8:**
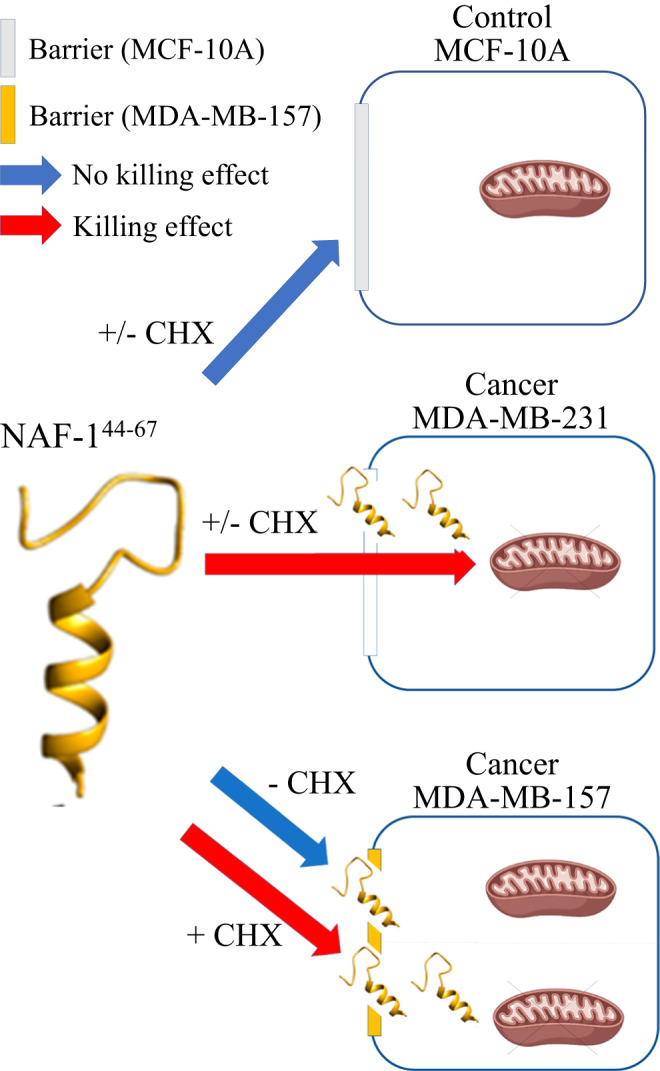
A hypothetical model for the effects of the NAF-1^44–67^ peptide on MCF-10A, MDA-MB-231, and MDA-MB-157 cells in the presence or absence of cycloheximide. The barrier of control MCF-10A cells to the peptide is shown to be unaffected by cycloheximide (CHX) and these cells are resistant to the peptide in the presence or absence of CHX. In contrast, MDA-MB-231 cells cannot block the peptide from entering and are susceptible to the peptide in the presence or absence of CHX. In the absence of CHX, MDA-MB-157 cells can block the peptide from entering and are therefore more resistant to it than MDA-MB-231 cells. However, in the presence of CHX, MDA-MB-157 cells are unable to block the peptide from entering and are sensitive to it. The model supports the use of a cell penetrating peptide-antibiotic/chemotherapy combination treatment against primary and metastatic cancer cells. CHX cycloheximide, NAF-1 nutrient autophagy factor 1.

Cancer cells can become resistant to different drugs/peptides through multiple routes (sometimes referred to as ‘escape’ mechanisms). These could involve blocking the entry of the drug/peptide, enhancing its active export, chemically modifying/degrading it inter- or intra-cellularly, partitioning/sequestering the drug/peptide away from its target, mutating, modifying, or eliminating the drug/peptide target, and/or bypassing the drug/peptide target function by activating alternative pathways. These mechanisms give rise to metastatic, drug-resistant, cancers, such as some of the ones addressed in this study (e.g., those represented by the MDA-MB-231 and MDA-MB-157 cell lines), that are associated with poor prognosis. Using novel drug combination therapies, such as the combination of CHX and NAF-1^44–67^, proposed by this study (Fig. 8), is therefore critical to our success in fighting metastatic cancers.

NEET proteins play a key role in the regulation of iron, iron-sulfur, and reactive oxygen species (ROS) metabolism and signaling in cancer cells [25–29]. Recently, a CPP derived from the NEET protein CISD2/NAF-1 (NAF-1^44–67^) was shown to have promising anticancer activity against MDA-MB-231 cells and tumors in both in vitro and in vivo model systems [15, 16]. Here we show that NAF-1^44–67^ is also effective against several other types of breast cancer cells, but that one type, MDA-MB-157, is resistant to it (Figs. 1, 2, and 6). Like MDA-MB-231, MDA-MB-157 is classified as triple-negative/basal-B mammary carcinoma breast cancer cell line. The two cell lines show significant similarities (e.g., [30–32]), as well as differences (e.g., [19, 33–36]), in their responses to chemotherapy agents, molecular mechanism function, and even membrane composition. Here we show that they are also different in their degree of susceptibility to the NAF-1^44–67^ peptide (Figs. 1, 2, and 6). We further show that this difference could result from differences in vesicle content, and/or the presence of PM protrusions, between MDA-MB-231 and MDA-MB-157 (Figs. 3 and 7 and Movies S1–S3). The high membrane activity of MDA-MB-157 (Figs. 3 and 7 and Movies S1–S3), that is likely accompanied by a higher rate of membrane turnover and/or a higher overall volume of membranes, could therefore prevent the NAF-1^44–67^ peptide from entering cells (Figs. 4–7). We further show that treatment of MDA-MB-157 cells with the protein synthesis inhibitor CHX (100 μM) that decreases vesicle content, as well as rate of extension and overall length of cellular protrusions, renders these cells susceptible to the peptide (Figs. 5–7). In support of the possibility that the membrane structures of MDA-MB-157 play an important role in protecting these cells from the peptide is also the finding that a higher dose of the peptide can negatively affect (or ‘break’) the resistance of MDA-MB-157 cells to the peptide (Fig. 2E), potentially a result of the peptide starting to saturate the cell membrane barriers and eventually entering cells. Our study therefore supports a model in which the NAF-1^44–67^ peptide (15 μM) can bind to the membranes of MDA-MB-157 cells, but cannot enter these cells, unless CHX (100 μM) is added (Figs. 4–6 and 8).

While combinations of CPPs and an antibiotic were shown to have a positive effect on the antimicrobial activity of CPPs (e.g., [37–40]), very little is known about the potential anticancer activity of combining CPPs with an antibiotic such as CHX. As the mechanisms underlying the effects of the protein synthesis inhibitor CHX on the efficacy of the NAF-1^44–67^ peptide, appear to involve suppressing the formation of PM protrusions and/or vesicle content (Fig. 7), it is important to note that PM protrusions are associated with enhanced metastatic activity of cancer cells. Increased PM protrusions elicited by growth factor stimulation was shown to play a critical role in the dissemination and metastasis of solid tumors (e.g., [41]), and tubulin-enriched membrane protrusions were shown to facilitate tumor cell reattachment to endothelial layers (e.g., [42]). As these structures could present a physical barrier for treatments with CPPs (especially energy-independent CPPs like NAF-1^44–67^) [15, 16], the potential solution presented in this work, of combining CPPs with an antibiotic protein synthesis inhibitor (NAF-1^44–67^ + CHX; Figs. 5–7), could represent a viable combination therapy that enhances the efficacy of CPPs towards metastatic cancers (Fig. 8). In addition to protein synthesis inhibitors such as CHX, several different chemotherapy agents that target the active metabolic rate of cancer cells could have a similar effect, enhancing the killing effect of CPPs like NAF-1^44–67^. Our findings therefore propose a new approach of using a combination of a CPP with an antibiotic protein synthesis inhibitor/chemotherapy agent, to target metastatic cancer cells that have a high PM activity (i.e., high content of vesicles and PM protrusions), that could physically shield them from certain CPPs (Fig. 8).

Although our results support a model in which the protein synthesis inhibitor treatment suppresses the formation of PM physical barriers against the peptide (Figs. 5–8; also supported by the effect of a higher dose of the peptide; Fig. 2E), we cannot rule out the possibility that CHX inhibited the synthesis of certain anti apoptosis/ferroptosis proteins that are unique to MDA-MB-157 cells, decreased the content of certain membrane-associated proteins involved in the binding/penetration of the peptide into cells, decreased the rate of active sequestrations, expulsion, or degradation of the peptide, and/or altered the membrane composition of these cells, decreasing their resistance to the NAF-1^44–67^ peptide. Given the energy-independent mode of NAF-1^44–67^ penetration into cells [15, 16], the dose effect of the peptide on MDA-MB-157 cells, suggesting a breaking of resistance at higher concentrations (Fig. 2), the fact that the peptide was detected attached to the PM of MDA-MB-157 cells in the presence or absence of CHX (Fig. 5), and the fact that the peptide did not cause damage to the mitochondria of MDA-MB-157 (in the absence of CHX; Fig. 4), we believe that a membrane structural barrier effect is a more plausible explanation for the effect of the protein synthesis inhibitor CHX on the resistance of MDA-MB-157 to NAF-1^44–67^ (Fig. 8). NAF-1^44–67^ is therefore able to bind to the membranes of MDA-MB-157, but it cannot enter these cells, unless CHX is added (Fig. 8). Our findings further support a model in which a cooperative process (i.e., multiple peptides acting together), or a very strong sequestration process by MDA-MB-157 cells (to the point of saturation), impact the killing effect of the peptide on MDA-MB-157 cells. Further studies are of course needed to refine our understanding of the mode of function of NAF-1^44–67^ + CHX (or NAF-1^44–67^+chemotherapy) combination treatments, their efficacy in in vivo model systems, and the molecular mechanisms underlying them. The results presented in the current study support future efforts into these studies, as well as promote the concept of using a CPP and protein synthesis inhibitor/chemotherapy combination therapy as a drug combination treatment for different metastatic epithelial cancers. In addition, they may also help to pinpoint possible origins for the selectivity of other peptides in cancer treatment.

## Materials and methods

### Cells cultures

All cell lines were received from the American Type Culture Collection (ATCC https://www.atcc.org/; Table S1). Hs 578 T was grown in DMEM HG 10% Fetal Bovine Serum (FBS) from Sigma (https://www.sigmaaldrich.com) plus Penicillin-Streptomycin (10,000 U/ml; Gibco/ThermoFisher https://www.thermofisher.com). BT-549, MDA-MB-231, MDA-MB-436, MDA-MB-157, and MDA-MB-453 were grown in RPMI-1640 10% FBS plus Penicillin-Streptomycin (10,000 U/ml; Gibco/ThermoFisher https://www.thermofisher.com). MCF-10A was grown according to ATCC procedures with 10% FBS and (100,000 U/ml Penicillin/Streptomycin). All cells were grown at 37 °C in a humidified Incubator at 5% CO_2_ (Binder-www.binder-world.com).

### Peptide

The NAF-1^44–67^ peptide FLGVLALLG(Y)LAVRP(F)LPK(K)KQQK; parenthesis correspond to the D enantiomer form of the amino acid) was synthesized by EZ Biolabs (http://www.ezbiolab.com). The NAF-1^44–67^ peptide was prepared from powder at a stock concentration of 200 µM in sterile saline. Final concentrations were reached in the appropriate media prior to each experiment [15].

### Cell death measurement

15,000 cells were plated per well and allowed to grow in a 96 well plate for three days prior to the experiments (in their appropriate media as described above). The day of the measurements, the media was replaced with phenol red free DMEM with 100,000 U/ml Penicillin/Streptomycin and Propidium iodide (PI; Excitation 531 nm emission 593 nm; Invitrogen/ThermoFisher; https://www.thermofisher.com) at a concentration of 0.1 mg/ml in the presence or absence of the NAF-1^44–67^ peptide at concentrations of 0, 5, 10, 15, or 30 μM of the NAF-1^44–67^ peptide. Cells were then imaged and quantified for cell death overtime using a BioTek Lionheart FX (BioTek; https://www.biotek.com) apparatus at 37 °C, 5% CO_2_ under humidified conditions.

### Vesicles labeling

In a 96 well plate, 15,000 cells were plated per well and allowed to grow for three days prior to the experiments. Cells were incubated with 5 µM of SynaptoRed^TM^ C2 in DMEM cell culture media for 1 h then washed in DMEM cell culture media, and vesicles were imaged using the BioTek Lionheart FX (https://www.biotek.com) at 37 °C, 5% CO_2_ under humidified condition. Excitation 596 nm and emission 615 nm were used. Image analysis and quantification were conducted using ImageJ (https://www.nih.gov).

### Peptide localization

The NAF-1^44–67^ peptide was labeled with 5(6)-carboxyfluorescein at its N-termini as described in [15]. In a 96 well plate, 15,000 cells per well were allowed to grow for three days prior to experiments. Vesicles and PM were labeled with 5 µM of SynaptoRed^TM^ C2 in DMEM cell culture medium for 1 h and washed with DMEM cell culture media. The cells were then incubated in phenol red free DMEM with 100,000 U/ml Penicillin/Streptomycin with 15 µM of the peptide (Fl-NAF-1^44–67^). The cells were imaged using a BioTek Lionheart FX (https://www.biotek.com) apparatus at 37 °C, 5% CO_2_ under humidified condition using the following wavelengths 586/647 nm for the SynaptoRed^TM^ C2 and 469/525 nm for Fl-NAF-1^44–67^. Image analysis and quantification were conducted using ImageJ (https://www.nih.gov). Briefly, the fluorescence signal of Fl- NAF-1^44–67^ peptide was overlapped with phase contrast images and the intensity of signal was then discriminated between PM and intracellular localization.

### Cell velocity and protrusions measurements

15,000 cells per well were allowed to grow in a 96 well plate for three days prior to experiments. The cells were imaged for 5 h using the BioTek Lionheart FX (BioTek; https://www.biotek.com) at 37 °C, 5% CO_2_ under humidified condition. Cell movements, protrusion length and formation rate were analyzed using ImageJ (https://www.nih.gov) form phase contrast images captured over time laps of 12 h with interval of 5 min.

### Transmission electron microscope (TEM)

All reagents for fixing the cells and TEM analysis were obtained from the Electron Microscopy Core at the University of Missouri (https://research.missouri.edu/electron-microscopy). Cells, plated at a density of 500,000 cells per well, were incubated in the presence or absence of the NAF-1^44–67^ peptide (15 µM). At 0 and 6 h post peptide application, the media was removed to collect any cells that may have detached. These cells were spun down at 1200 rpm and fixed in 2% paraformaldehyde, 2% glutaraldehyde in 100 mM sodium cacodylate buffer at pH 7.35 (detached cells). The remaining (adhered cells) were rinsed once in Phosphate Buffered Saline (PBS) and fixed in 2% paraformaldehyde, 2% glutaraldehyde in 100 mM sodium cacodylate buffer of pH 7.35 for 1 h. Cells were then gently scraped off the plate and allowed to remain in the fixative buffer for another 30 min. The cells were then removed, and the plates rinsed several times with fixative. The detached, fixed, and washed cells were added to the adhered fixed cells and cells were subjected to further processing and imaging as described in [15] using the JEOL JEM 1400 transmission electron microscope (JEOL, Peabody, MA, USA). Images were acquired at 80 kV using a Gatan Rio CMOS camera (Gatan, Inc, Pleasanton, CA), and quantification was conducted using ImageJ (https://www.nih.gov).

### Image and statistical analysis

Images generated by the BioTek Lionheart FX (BioTek; https://www.biotek.com/) apparatus were processed using the manufacturer analysis software BioTek Gen 5 version 3.08.01. The data obtained was exported in excel format and analyzed using Graph Pad software version 9.4.1. ANOVA tests were performed using the GraphPad software version 9.

### Supplementary information

Table S1

List of Suppl Material

Author contribution (also appears in the manuscript file)

Supplementary Movie 1

Supplementary Movie 2

Supplementary Movie 3

Supplementary Movie 4

Supplementary Movie 5

Supplementary Movie 6

Supplementary Movie 7

Supplementary Movie 8

Supplementary Movie 9

Supplementary Movie 10

Supplementary Movie 11

Supplementary Movie 12

## Data Availability

All data are included in the manuscript and Supplementary Material.
