# Supplementary material for: A combination of a cell penetrating peptide and a protein translation inhibitor kills metastatic breast cancer cells
Source: Cell Death Discov. 2023 Aug 31;9:325. doi: 10.1038/s41420-023-01627-3 (PMC10471752; doi:10.1038/s41420-023-01627-3)
Supplement: Supplementary file 1 — Table S1 [file 41420_2023_1627_MOESM1_ESM.pdf]

Table S1. Epithelial breast cancer cell lines used in the study.

Subtype2	Name	Histology	Tumor source	MutantGene	Zygosity	Gene Sequence
M	BT-549	Ductal carcinoma	primary	PTEN	homozygous	c.823delG
				RB1	homozygous	c.265_607del343
				TP53	homozygous	c.747G>C
MSL	Hs 578T	Carcinoma	primary	CDKN2A	homozygous	c.1_471del471
				HRAS	heterozygous	c.35G>A
				PIK3R1	homozygous	c.1358_1359insTAA
				TP53	homozygous	c.469G>T
	MDA-MB-231	Adenocarcinoma	metastasis, pleural effusion	BRAF	heterozygous	c.1391G>T
				CDKN2A	homozygous	c.1_471del471
				KRAS	heterozygous	c.38G>A
				NF2	homozygous	c.691G>T
				TP53	homozygous	c.839G>A
	MDA-MB-436	Adenocarcinoma	metastasis, pleural effusion	BRCA1	homozygous	c.5277+1G>A
				RB1	homozygous	c.607_608ins227
	MDA-MB-157	Medulallary carcinoma	metastasis, pleural effusion	NF1	homozygous	c.8253_8268del16
				TP53	homozygous	c.261_286delAGCCCCCTCCTGGCCCCTGTCATCTT
LAR	MDA-MB-453	Carcinoma	metastasis, pleural effusion	CDH1	homozygous	c.1913G>A
				PIK3CA	heterozygous	c.3140A>G
