# Supplementary material for: A combination of a cell penetrating peptide and a protein translation inhibitor kills metastatic breast cancer cells
Source: Cell Death Discov. 2023 Aug 31;9:325. doi: 10.1038/s41420-023-01627-3 (PMC10471752; doi:10.1038/s41420-023-01627-3)
Supplement: Supplementary file 2 — List of Suppl Material [file 41420_2023_1627_MOESM2_ESM.pdf]

List of Supplementary Material

Table S1. Epithelial breast cancer cell lines used in the study.

Movie S1. Untreated MCF-10A cells

Movie S2. Untreated MDA-MB-231 cells

Movie S3. Untreated MDA-MB-157 cells

Movie S4. MDA-MB-157 cells treated with NAF-1^{44-67} (15 μM) in the presence of PI

Movie S5. MDA-MB-157 cells treated with CHX (100 μM) in the presence of PI

Movie S6. MDA-MB-157 cells treated with NAF-1^{44-67} (15 μM) + CHX (100 μM) in the presence of PI

Movie S7. MCF-10A cells treated with NAF-1^{44-67} (15 μM) in the presence of PI

Movie S8. MCF-10A cells treated with CHX (100 μM) in the presence of PI

Movie S9. MCF-10A cells treated with NAF-1^{44-67} (15 μM) + CHX (100 μM) in the presence of PI

Movie S10. MDA-MB-231 cells treated with NAF-1^{44-67} (15 μM) in the presence of PI

Movie S11. MDA-MB-231 cells treated with CHX (100 μM) in the presence of PI

Movie S12. MDA-MB-231 cells treated with NAF-1^{44-67} (15 μM) + CHX (100 μM) in the presence of PI
