# Supplementary material for: A combination of a cell penetrating peptide and a protein translation inhibitor kills metastatic breast cancer cells
Source: Cell Death Discov. 2023 Aug 31;9:325. doi: 10.1038/s41420-023-01627-3 (PMC10471752; doi:10.1038/s41420-023-01627-3)
Supplement: Supplementary file 3 — Author contribution (also appears in the manuscript file) [file 41420_2023_1627_MOESM3_ESM.pdf]

Author contribution

L.R, H-B.M., D.G., and O.K. performed the experiments and analyzed the data. R.M., H-B.M., L.J.W., A.F., R.N., and R.E. wrote the manuscript.
